# Fatty acid metabolism and colon cancer protection by dietary methyl donor restriction

**DOI:** 10.1007/s11306-021-01831-1

**Published:** 2021-09-03

**Authors:** Oladimeji Aladelokun, Matthew Hanley, Jinjian Mu, John C. Giardina, Daniel W. Rosenberg, Charles Giardina

**Affiliations:** 1 Center for Molecular Oncology, University of Connecticut Health Center, The University of Connecticut School of Medicine, 263 Farmington Ave., Farmington, CT 06030-3101 USA; 2 Statistical Consulting Services, University of Connecticut, Storrs, CT USA; 3 Center for Health Decision Science, Harvard T.H. Chan School of Public Health, Boston, MA USA; 4 Department of Molecular and Cellular Biology, University of Connecticut, Storrs, CT USA

**Keywords:** Dietary methyl donors, Colon cancer, Plasma biomarkers, Carnitine, Fatty acid metabolism

## Abstract

**Introduction:**

A methyl donor depleted (MDD) diet dramatically suppresses intestinal tumor development in *Apc*-mutant mice, but the mechanism of this prevention is not entirely clear.

**Objectives:**

We sought to gain insight into the mechanisms of cancer suppression by the MDD diet and to identify biomarkers of cancer risk reduction.

**Methods:**

A plasma metabolomic analysis was performed on *Apc*^*Δ14/*+^ mice maintained on either a methyl donor sufficient (MDS) diet or the protective MDD diet. A group of MDS animals was also pair-fed with the MDD mice to normalize caloric intake, and another group was shifted from an MDD to MDS diet to determine the durability of the metabolic changes.

**Results:**

In addition to the anticipated changes in folate one-carbon metabolites, plasma metabolites related to fatty acid metabolism were generally decreased by the MDD diet, including carnitine, acylcarnitines, and fatty acids. Some fatty acid selectivity was observed; the levels of cancer-promoting arachidonic acid and 2-hydroxyglutarate were decreased by the MDD diet, whereas eicosapentaenoic acid (EPA) levels were increased. Machine-learning elastic net analysis revealed a positive association between the fatty acid-related compounds azelate and 7-hydroxycholesterol and tumor development, and a negative correlation with succinate and β-sitosterol.

**Conclusion:**

Methyl donor restriction causes dramatic changes in systemic fatty acid metabolism. Regulating fatty acid metabolism through methyl donor restriction favorably effects fatty acid profiles to achieve cancer protection.

**Supplementary Information:**

The online version contains supplementary material available at 10.1007/s11306-021-01831-1.

## Introduction

Folate consumption and one-carbon metabolism (OCM) are essential for numerous biosynthetic processes, including nucleotide and amino acid synthesis. OCM can also impact cellular epigenetics by providing methyl donors for DNA and histone methylation (Bistulfi et al., 2010; Hanley & Rosenberg, 2015; Bao et al., 2016; Miyo et al., 2017; Noguchi et al., 2018). Epidemiological studies aimed at determining the relationship between dietary folate intake and colorectal cancer (CRC) have generated conflicting results (Chae & Yun, 2007; Cole et al., 2007). Evidence from observational studies pointed to an inverse relationship between dietary folate intake and CRC risk (reviewed in Mahmoud & Ali, 2019). However, a large polyp prevention study revealed an adenoma-promoting effect of folate supplementation in patients with a history of these lesions: individuals administered folate (1 mg/d) showed a > 40% increased risk of colon adenoma development compared to placebo (Cole et al., 2007). Based on this study, it has been suggested that while folate can help maintain a healthy colonic epithelium, it may promote the growth and progression of established pre-cancerous lesions in the colon. These findings are of particular importance given the wide-spread fortification of grain products (such as breads and cereals) with folate that was originally instituted to reduce the occurrence of birth defects associated with folate deficiency (Centers for Disease & Prevention, 2004).

Preclinical studies in mouse CRC models have supported the tumor-promotional effects of high dietary folate (Van Guelpen et al., 2006; Gylling et al., 2014; Alves da Silva et al., 2019). Song and colleagues showed that increased dietary folate intake promoted colon tumor development in *Apc*^*Min/*+^ mice after neoplastic crypts had formed (Song et al., 2000). These mice undergo loss of heterozygosity in the tumor suppressor gene, *Apc* and spontaneously develop multiple intestinal neoplasia (Moser et al., 1995). This genetic model recapitulates the events associated with colon tumor development in patients with familial adenomatous polyposis. The *Apc* gene is also mutated in most sporadic human colorectal cancers (Fearon, 2011). In addition, lowering folate was found to have a protective effect; a study by Lawrance and Rozen reported that low folate status suppressed tumor formation in *Apc*^*Min/*+^ mice (Lawrance et al., 2009). Our laboratory previously reported that combined dietary restriction of the methyl donors folate, methionine, choline, and vitamin B12, produced a dramatic reduction in the number and size of intestinal tumors in two *Apc*-driven mouse models of colorectal cancer (Kadaveru et al., 2012; Hanley et al., 2016). Our findings are consistent with the promotional effects of OCM at later stages of cancer progression (Kim, 2004, 2006; Van Guelpen et al., 2006; Alves da Silva et al., 2019). We also reported that the cancer prevention associated with methyl donor deficient (MDD) diet persists even beyond dietary repletion of methyl donors (Hanley et al., 2016, 2020), indicating a stable, long-lasting cancer-protective activity.

We are interested in understanding which metabolic changes caused by the MDD diet are responsible for reducing CRC risk. In our recent metabolomics analysis of the colonic mucosa, we found that tumor protection in *Apc*^*Δ14/*+^ mice by the MDD diet was associated with a reduction in carnitine and acylcarnitines in colon tissue (Hanley et al., 2020). Carnitine is utilized by cells to transport fatty acids into the mitochondria for oxidation and energy production. It is produced by a number of different tissues, most notably the liver and kidney. Carnitine is also utilized for fat uptake by the small intestine (Leichter et al., 1987). We therefore wished to determine whether the MDD diet reduced systemic carnitine synthesis and fatty acid uptake. Overall, we found reductions in circulating carnitine, acylcarnitines and fatty acids by the MDD diet. Interestingly, plasma metabolites related to fatty acid metabolism were found to correlate most closely with tumor incidence, suggesting that this metabolic change underlies cancer protection. We discuss how the plasma changes might relate to cancer risk in the colon and how the metabolites identified might serve as potential biomarkers for CRC risk.

## Methods

### Diet administration and sample collection

Four-week-old *Apc*^*Δ14/*+^ mice, harboring a mutation within the *Apc* tumor suppressor gene, were randomized into 4 experimental groups. One group received the MDS diet (Harlan-Teklad) containing an adequate amount of folate, choline, methionine and vitamin B12 for a total of 18 weeks. The MDD group received the experimental MDD diet deficient for folate, choline, methionine and vitamin B12 (Harlan-Teklad). Pair-fed (PF) mice were pair-fed the MDS diet equivalent to the amount that was consumed in the MDD group. A pilot study shows that the MDD mice consumed less food than the MDS mice weekly. Due to the roles of caloric restriction in modulating cancer risk outcomes, we included a fourth group, the Pair-Fed (PF) group to control for the possible roles caloric restriction may play in tumor multiplicity in the *Apc*-mutant mice. The weight of food consumed by the MDD group was used to determine how much food to provide to the MDS Pair-Fed group.

To understand the effects of temporary methyl donor deficiency, a ‘repletion group’ (MDDR) received the MDD diet for 11 weeks and were then returned to the MDS control diet for an additional 7 weeks. Body weight was monitored weekly during the diet administration. Both male and female mice were used in this study and all mice were housed at a constant temperature at 22 °C and allowed free access to drinking water. Mice were euthanized at 22 weeks and evaluated for tumor development (Giardina et al., 2015). At the time of euthanasia, blood was withdrawn from the heart using a pre-heparinized 18G needle into EDTA-coated tubes. The blood was centrifuged at 5000×*g* for 10 min at 4°. Plasma was separated and stored at − 80° until analysis. For colon metabolomic studies, normal-appearing segments of whole tissue were snap-frozen and stored at − 80 °C until downstream analysis.

### Metabolomics profiling

The metabolite profiling of the colon tissue samples have been previously described (Hanley et al., 2020). Four male (57%) and three female (43%) mice per group were selected for this plasma metabolomics analysis. Mouse plasma was collected after euthanasia and stored at − 80 °C until needed. Untargeted mass spectroscopy-based metabolomic profiling of *Apc*^*Δ14/*+^ mice plasma (n = 7 per group) was performed by Metabolon, Inc. (Durham, NC) as previously described (Hanley et al., 2020). The samples were selected based on size and body weight. Metabolomics analysis was conducted using three independent methods: a UPLC method for basic species using the Waters Acquity system (Waters, Millford), a UPLC/MS method optimized for acidic species and a GC/MS method for analyzing derivatized samples. Samples were thawed on ice before the extraction process. Extraction was executed using the automated MicroLab STAR® (Hamilton Robotics). Proteins were removed by precipitation with methanol under vigorous shaking for 2 min (Glen Mills GenoGrinder 2000) followed by centrifugation for 5 min at 700×*g*. The extraction process ensured the removal of proteins, small molecules bound to protein or trapped in protein matrix precipitates. The recovery extract was divided into four fractions: one fraction was reconstituted in 6.5 mM ammonium bicarbonate (pH 8) and was subject to analysis by UPLC-MS/MS (OrbiElite, Thermo Scientific) with positive ion mode electrospray ionization. A second fraction was reconstituted in 0.1% formic acid and was subject to analysis by UPLC-MS/MS (Thermo Scientific) with negative ion mode electrospray ionization. Both fractions were stored overnight under nitrogen and under dried vacuum overnight. A third fraction was subject to treatment with BSTFA and 1% trimethylchlorosilane in cyclohexane/dichloromethane/acetonitrile (5:4:1) and 5% triethylamine. Each sample was dried under vacuum overnight before analyzing with GC/MS (Dual Stage Quadrapole system, Thermo Scientific). A fourth fraction was stored at − 80 °C for rerun purposes if necessary. A detailed protocol of the UPLC, UPLC/MS and GC/MS methods is previously outlined (Hanley et al., 2020) and described elsewhere (Guo et al., 2015).

#### Quality control, compound identification and data curation

Technical replicates of pooled plasma sample aliquots (described above) served as a quality control (QC) to determine process variability, calculated by the median relative standard deviation (RSD) for all endogenous metabolites (non-instrument standards) present in each sample.

Identification of known chemical entities was based on comparison to chemical standard entries of an in-house metabolomic library that included retention time, mass-to-charge ratio (m/z), preferred adducts, and in-source fragments as well as the associated MS/MS spectra for all the molecules in the library. Known metabolites were curated by comparing the individual spectra to standard reference libraries using the Quantify Individual Components in a Sample termed QUICS, a chemo-centric method that reduces the number of false discoveries. (Evans et al., 2009; Dehaven et al., 2010).

### Bioinformatics

Visualization of plasma metabolomics data was conducted in R (version 3.6.1, R Core Team). A log transformation was applied to account for heteroscedasticity and correct for skewed data distribution (Jauhiainen et al., 2014). Auto-scaling, normalization of data and pathway analysis were performed using the online metabolomics analysis tool, Metaboanalyst 4.0 that includes an updated KEGG pathway database (http://www.genome.jp/kegg/) (van den Berg et al., 2006; Jauhiainen et al., 2014; Chong et al., 2019).

### Statistical analysis

For the plasma metabolomics analysis, statistical significance was assessed by one-way analysis of variance (ANOVA). As indicated in the Supplementary Data, statistical significance was assessed for the ANOVA results using a cutoff of 0.0083 after applying the Bonferroni correction to adjust for multiple comparisons (six hypotheses were tested with α = 0.05).

Data that are not normally distributed were analyzed using non-parametric Kruskal–Wallis test.

All groups marked with “a” have a statistically significant difference compared to groups marked with “b” or “c” or “d”.

A principal component analysis (PCA) was used to visualize groups based on differences in metabolites scaled intensity. Statistical analyses were performed using GraphPad Prism 8 software (GraphPad Software, Inc., La Jolla, CA, USA).

Elastic net regularized regression (Kirpich et al., 2018) was used to select important metabolites that are most closely associated with colon tumor burden. The diet group assignment was not considered in this portion of the analysis. This method was selected for its ability to handle data where the number of samples is smaller than the number of predictors (metabolites). The elastic net regularization method is a combination of Lasso (least absolute shrinkage and selection operator) and ridge regression, and can be used for variable selection in the presence of multicollinearity problems (Zou, 2005). The mixing and regularization parameters for the elastic net (often denoted as alpha and lambda, respectively) were selected using leave-one-out cross-validation (CV) with the cv.glmnet function in the R package glmnet (Friedman et al., 2010). We assumed a Poisson distribution with a log link in order to model the tumor count as a function of standardized metabolite levels and the optimal alpha and lambda values were selected to minimize the average out-of sample deviance across the cross-validation folds. In using the elastic net model, the goal is to identify metabolites that are good predictors of colon tumor counts, which might potentially be linked with tumor development.

## Results

### The tumor-preventing MDD diet alters plasma levels of one-carbon metabolites

**Fig. 1:**
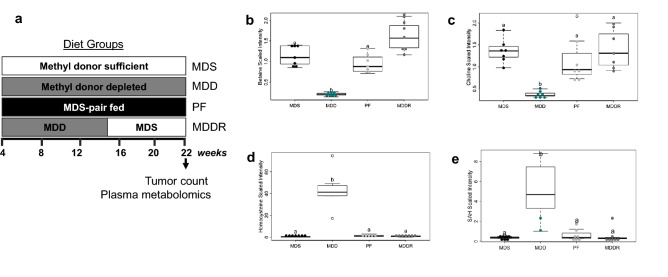
Study design and changes in the levels of metabolites involved in one-carbon metabolism. **a** Four-week-old *Apc*^*D14*^ mice, harboring a mutation within the *Apc* tumor suppressor gene, were randomized into 4 experimental groups. Group I received the methyl donor sufficient (MDS) diet containing an adequate amount of folate, choline, methionine and vitamin B12. Group II received the experimental methyl donor deficient (MDD) diet. Group III mice were pair-fed (MDS-PF) the MDS diet in equivalent amount as Group II. The weight of food consumed by the MDD group was used to determine how much food to provide to the MDS Pair-Fed group. Group IV mice were maintained on the MDD diet for 14 weeks, before repletion of dietary methyl donors (MDDR). **b** Levels of betaine (− 5.46-fold, p < 0.001), **c** choline (− 3.7-fold, p < 0.001) were reduced under conditions of dietary methyl restriction. **d** MDD diet causes a significant increase in the levels of homocysteine (14.3-fold, p < 0.002) and **e** S-adenosylhomocysteine (88.7-fold, p < 0.001). Statistically significant differences (p < 0.05) between groups are indicated by letters placed above each group. “MDS” = MDS ad libitum*,* “MDD” = Methyl donor deficient, “PF” = MDS pair-fed, and “MDDR” = MDD:MDS-Repletion group. All groups marked with “a” have a statistically significant difference compared to groups marked with “b”

**Fig. 2:**
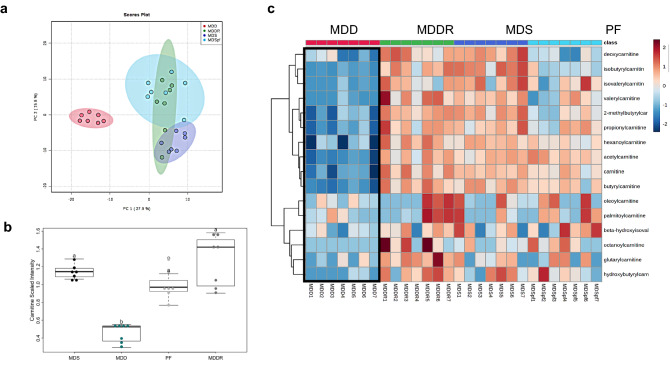
Distinct plasma metabolome and altered levels of metabolites associated with fatty acid oxidation. **a** PCA plot shows a distinct metabolic profile associated with dietary MDD. A partial overlap of the MDDR samples with both MDS ad libitum and MDS PF control groups indicates metabolic changes that persist at least 7 weeks beyond active methyl donor repletion. **b** The MDD diet causes a significant reduction in the level of plasma carnitine (− 3.61-fold, p < 0.05) suggesting inhibition of FAO. All groups marked with “a” have a statistically significant difference compared to groups marked with “b”. **c** Heatmap depicting the relative concentration of plasma metabolites associated with fatty acid β-oxidation (FAO) using normalized intensities of sixteen (16) significant metabolites (p < 0.05). The heatmap was constructed by applying Euclidean distance and Ward’s methods to measure the absolute distance between the points in space and metabolite clustering, respectively. These results were obtained from seven animals in each of the four diet groups

Plasma samples derived from a previous study (Hanley et al., 2020) determining the effect of a reduced methyl donor diet on cancer risk were analyzed. This study is shown schematically in Fig. 1a. As previously described, the MDD diet was associated with a reduction in intestinal tumor multiplicity relative to MDS. MDS-PF was also associated with a reduction in tumor multiplicity compared to MDS, though the effect was smaller than MDD. MDD-associated reductions persisted for at least 7 weeks beyond repletion of methyl donors (MDDR). This earlier study shows that dietary manipulation of methyl donor nutrients impacts colon cancer risk in the *Apc*^*Δ14/+*^ mouse model.  To further study the metabolic changes associated with methyl donor depletion, we used the Metabolon metabolomics platform to quantify changes in metabolite concentrations in *Apc*^*Δ14/*+^ mouse plasma. Plasma OCM metabolites were altered as anticipated, with decreased levels of choline, the choline derivatives betaine and dimethylglycine, and cystine (a methionine derivative) (Fig. 1b–e; Supplemental Fig. 1a, b). This was accompanied by an increase in the plasma levels of homocysteine and S-adenosylhomocysteine (SAH) in the MDD fed mice (Fig. 1d, e). Principal component analysis of the metabolomics data showed that the MDD mice formed a distinct cluster, consistent with the more pronounced dietary difference of this group (Fig. 2a). The overlap between the MDDR group and the MDS control groups shows the reversibility of the methyl donor depletion.

### The MDD diet decreases plasma carnitine, acylcarnitines and fatty acids

In our previous analysis, the MDD diet induced a dramatic drop in colon carnitine levels. Analysis of the plasma samples likewise showed a reduction in the level of circulating carnitine in the MDD group (Fig. 2b). This reduction was also observed in all acylcarnitine species detected (Fig. 2c). The dramatic reduction in carnitine was reversible by methyl donor repletion. Pair feeding (MDS PF) was associated with a small and non-significant reduction on plasma carnitine level, suggesting that methyl donor deficiency is the primary cause of this effect (Fig. 2c). This data indicates that there is a systemic drop in carnitine synthesis, consistent with changes in liver and kidney carnitine synthesis.

**Fig. 3:**
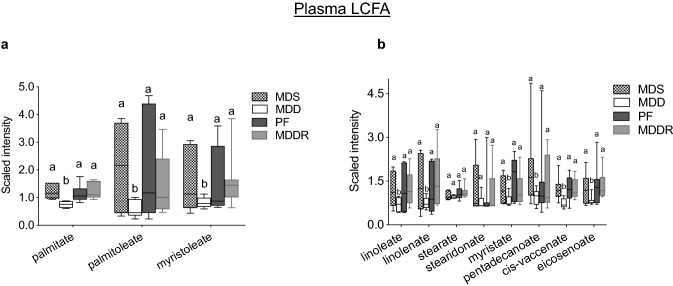
Modulation of plasma long-chain fatty acids (LCFAs). **a** The MDD diet caused a reduction in the levels of palmitate (− 2.0-fold, p < 0.003), palmitoleate (− 3.6-fold, p < 0.03), myristoleate (− 2.0-fold, p < 0.05). **b** A panel of LCFAs including linoleate, linolenate, myristate, cis-vaccinate and eicosanoate was reduced under conditions of dietary methyl donor restriction. All groups marked with “a” have a statistically significant difference compared to groups marked with “b”

Impaired carnitine synthesis limits fatty acid import into the mitochondria for β-oxidation. In addition, carnitine is used to import fatty acids into the endoplasmic reticulum of enterocytes for chylomicron assembly and fat absorption (Washington et al., 2003). Consistent with a reduced fat absorption in MDD mice, Fig. 3a shows reduced levels of the major plasma fatty acid species palmitate, palmitoleate and myristoleate. A range of other fatty acids including linoleate and linolenate, myristate, pentadecanoate, cis-vaccenate and eicosenoate were significantly reduced (Fig. 3b). Although most fatty acids show a large reduction, others are less effected; for example stearate and stearidonate are not significantly affected (Fig. 3b). Overall, these data indicate that the MDD diet limits fatty acid absorption, and that most but not all plasma fatty acid levels are reduced.

**Fig. 4:**
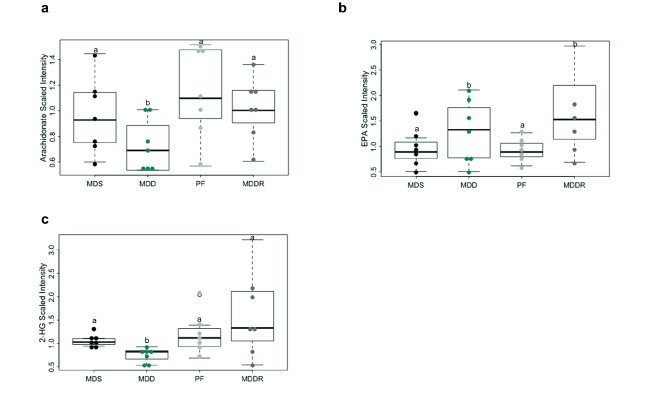
Changes in the levels of polyunsaturated fatty acids (PUFA) and oncometabolite 2-hydroxyglutarate (2-HG). **a** Methyl donor depletion causes a reduction in the levels of arachidonic acid (− 2.30, p < 0.04), a potential tumor-promoting metabolite. **b** Eicosapentaenoate (EPA) level was increased (1.33-fold) in MDD group and (2.0-fold, p < 0.013) in MDDR group indicating that the increased EPA level was sustained for at least seven weeks beyond dietary methyl donor repletion. **c** 2-hydroxyglutarate (2-HG), an oncometabolite was significantly reduced after methyl donor restriction (− 1.4-fold, p < 0.002. All groups marked with “a” have a statistically significant difference compared to groups marked with “b”

Given the effects of the MDD diet on fat absorption and fatty acid metabolism, we examined the levels of fatty acids and fatty acid metabolites associated with colon cancer development. Arachidonic acid and its prostaglandin metabolites are well-established promoters of colon cancer (Wang & Dubois, 2006). Consistent with the overall drop in plasma fatty acids, circulating arachidonic acid was significantly lower in MDD mice (Fig. 4a). We also found that the MDD diet reduced the expression of *Cox2* and *mPges1* in normal and cancer tissue (which are required for prostaglandin synthesis; Supplemental Fig. 2a–c). Interestingly, EPA, an alternative fatty acid substrate for *Cox2* that suppresses prostaglandin synthesis (Calviello et al., 2004) was increased in MDD-fed mice. Another fatty acid-derived metabolite implicated in colon cancer is 2-hydroxyglutarate, an oncometabolite that can induce epigenetic and metabolic reprogramming of cancer cells (Han et al., 2018). 2-hydroxyglutarate can be derived through the metabolism of hydroxybutyrate. 2-hydroxyglutarate levels were reduced by the MDD diet (Fig. 4c). These data point to possible factors contributing to cancer risk reduction in MDD mice.

### Effects of MDD on TCA intermediates

**Fig. 5:**
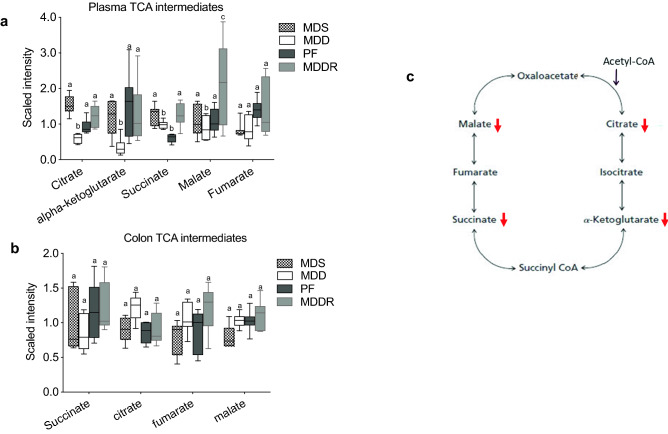
Methyl donor restriction alters metabolites involved in Kreb’s cycle. **a** A panel of metabolites involved in the tricarboxylic acid cycle was affected by the MDD diet. Levels of citrate (− 3.0-fold, p < 0.0001), ⍺-ketoglutarate (− 4.11-fold, p < 0.0001), succinate (− 1.3, p < 0.001), and malate (− 1.3-fold, p < 0.04) were reduced by methyl donor restriction. **b** In contrast, colon levels of succinate, citrate, fumarate and malate were not altered by MDD. **c** Schematics highlighting the affected metabolites that maintain the citric acid cycle flux. All groups marked with “a” have a statistically significant difference compared to groups marked with “b” and “c”. Similarly, all groups marked with “b” are significantly different relative to groups marked with “a” and “c”

Given the utilization of acylcarnitines for fatty acid uptake by the mitochondria, we determined whether the MDD diet affected components of the TCA pathway in the plasma and colon (Fig. 5). As shown in Fig. 5a, a reduction in the level of several TCA components was observed in the plasma. These reductions were more pronounced for intermediates found early in the cycle (citrate and ⍺-ketoglutarate), relative to metabolites later in the cycle (succinate, malate, and fumarate). This finding suggests that a reduction in fatty acid availability may reduce TCA activity to some degree. Interestingly, TCA pathway components were less effected in colon tissue, suggesting that this tissue relies on a different energy source to run the Krebs cycle, potentially luminal short chain fatty acids (Scheppach, 1994).

### Metabolites associated with tumor development

Given that we have tumor incidence numbers and plasma metabolite data for each of the 28 animals across the four different diet groups, we determined which plasma metabolites were most closely associated with tumor development. To the extent that the predictors selected by the elastic net method can be considered to be the most important variables in predicting the outcome, the metabolites selected by the method can be viewed as those with the strongest association with tumor development. We used the R package glmnet with leave-one-out cross validation to implement the method (Friedman et al., 2010). The optimal values of lambda and alpha, parameters that minimized the average deviance across the cross validation folds were 11.28 and 0.05 respectively. The metabolites selected by the method are those with the closest association with tumor development. The metabolites with the highest positive and negative associations are shown in Table 1 (all associated metabolites with non-zero coefficients from the elastic net regression are shown in Supplemental Table 1). Azelate and 7 α-hydroxycholesterol were both positively associated with tumor development. The two metabolites most associated with lower tumor incidence are β-sitosterol and succinate. β-sitosterol is a plant/diet derived phytosterol that reduces cholesterol up-take (Ikeda & Sugano, 1983). Although succinate is best known for its role in the TCA cycle (Connors et al., 2018), it has a number of other activities (Zhao et al., 2017; Connors et al., 2018). Whether these associations are mechanistically linked to tumor development is not clear, but some possibilities are addressed in the Discussion.

**Table 1:** Top four plasma metabolites identified as significant predictors of tumor count using elastic net and their corresponding coefficient values

Metabolite	Coefficient	MSI level*
Azelate	0.0878	1
7 α-hydroxycholesterol	0.0865	1
β-sitosterol	− 0.0864	1
Succinate	− 0.0959	1

*Metabolomics standards initiative (MSI) level is based on MSI guidelines for naming non-novel metabolites (Sumner et al., 2007). Selected metabolites in this dataset are classified as “Tier 1”

## Discussion

High levels of dietary methyl donors may establish a nutrient-rich condition that is unintentionally tumor-promoting, particularly for high-risk individuals prone to advanced adenoma development (Ryan & Weir, 2001). Conversely, limiting dietary methyl donors may be beneficial to high-risk patients. To determine the extent to which limitations in dietary methyl donors can suppress colon cancer, we designed the experimental MDD diet with stringent reductions in methyl donors. This MDD diet is strongly protective in mouse colon cancer models (Kadaveru et al., 2012; Hanley et al., 2016, 2020). Although this diet is unrealistic for humans, we have used it to study the metabolic changes linked to tumor suppression. Previous work showed a surprisingly strong reduction in colon carnitine levels, suggesting that fatty acid metabolism plays a role in tumor suppression. To better understand how this diet reduces cancer risk, we determined the effect of the MDD diet on the plasma metabolome, reasoning that this information could clarify mechanisms of action and provide plasma biomarkers for translational studies.

As found in the colon (Hanley et al., 2020), large reductions in carnitine and acyl-carnitines were observed in the plasma. This finding indicates that there is a systemic reduction in carnitine synthesis, likely by the liver and kidney. Carnitine synthesis (from lysine) requires SAM, so the effect of the MDD diet on its synthesis is likely a direct result of reduced methionine levels. Our previous study revealed that the MDD diet also reduced colonic methionine levels (Hanley et al., 2020). In both the plasma and colon, carnitine synthesis and the related effects on fatty acid metabolism are among the most dramatically impacted pathways, suggesting that this pathway is particularly sensitive to dietary methyl donor uptake. Other metabolic pathways, such as nucleotide synthesis, may take precedence in the context of limited methyl donor availability.

Carnitine attachment to fatty acids facilitates their transport into the mitochondria for oxidation. Reduced carnitine levels may therefore decrease activity of the Krebs cycle (and mitochondrial ATP synthesis). Whether this reduction is responsible for suppressing colon tumor formation is not clear, as we do not observe a reduction in Krebs cycle intermediates in the colon. However, more work needs to be done in this area, as measuring the steady state levels of Krebs cycle intermediates may not be a good indicator of pathway flux. In addition to its role in mitochondrial fatty acid uptake, fatty acid uptake in the intestine is also facilitated by carnitine (Leichter et al., 1987; Washington et al., 2003). Enterocytes in the small intestine make acyl-carnitines from absorbed fatty acids for transport into the ER and the assembly of chylomicrons (Abo-Hashema et al., 1999). Reduced fatty acid absorption appears to be a major effect of the MDD diet. Interestingly, some fatty acids are affected more than others, suggesting a hierarchy of uptake and metabolism. To assess the potential impact of these fatty acid changes on colon tumor incidence, we looked at fatty acids and fatty acid metabolites with known effects on cancer promotion. We found that arachidonic acid, the precursor of the potent tumor promoter PGE2, was reduced, whereas the tumor-suppressing fatty acid EPA was increased. Interestingly, EPA levels were also elevated in MDDR mice, suggesting that this effect persists following dietary methyl donor repletion. Finally, 2-HG, an oncometabolite that can be derived from hydroxybutyrate (Liesenfeld et al., 2016), was also reduced. Overall, the MDD diet appears to alter the spectrum of fatty acids in a manner favorable to colon cancer suppression, although the metabolic pathways underlying these changes are not yet clear.

To gain further insight into the relationship between diet and cancer risk, we employed elastic net regression to identify plasma metabolites most closely associated with tumor incidence. Identification of related metabolites can suggest potential mechanisms of cancer protection for further study and indicate potential biomarkers for translational studies. Azelate and 7 α-hydroxycholesterol were the top hits positively associated with tumor development. The positive association of azelate with cancer risk may be related to a higher level of fatty acid uptake. Azelate is formed by the peroxidation and cleavage of oleate (Rocchiccioli et al., 1986). The detection of azelate as a cancer risk marker over other fatty acids may be due to the fact that azelate is slowly metabolized, which may make it an effective marker of high fatty acid uptake (being a dicarboxylic acid, azelate is not efficiently oxidized by the mitochondria and relies largely on peroxisome metabolism) (Suzuki et al., 1989). Likewise, the tracking of 7 α-hydroxycholesterol with tumor incidence is consistent with fatty acid up-take contributing to cancer risk; primary bile acids derived from 7 α-hydroxycholesterol would promote lipid up-take from the diet. Previous work showed that the MDD diet decreases the level of secondary bile acids in the colonic mucosa, including tumor-promoting deoxycholate (Bernstein et al., 2011). The reduction in primary bile acid production may therefore decrease cancer risk by in part by reducing secondary bile acid production in the colon.

The two metabolites most closely associated with lower tumor incidence are β-sitosterol and succinate. β-sitosterol is a plant/diet-derived phytosterol that reduces cholesterol uptake and circulating LDL levels by competing with cholesterol for space within lipoprotein micelles (Fernandez & Vega-Lopez, 2005). The association between high β-sitosterol and lower cancer risk is in general agreement with the potential role of bile acids in cancer promotion discussed above. The relationship between succinate and lower cancer risk presents a more complex case. Although succinate is best known for its role in the TCA cycle, it has a number of other activities that could impact tumorigenesis (Dalla Pozza et al., 2020). Given that so many reactions utilize and produce succinate, additional work will be required to establish its role in mitigating cancer risk in this model.

This study and previous work point to carnitine synthesis and fatty acid uptake as playing a central role in determining colon cancer risk (Hanley et al., 2020). Although the diet used in these studies is not suitable for humans, identifying consequent metabolic changes as important determinants of cancer risk will facilitate the development of more targeted dietary alterations. The plasma biomarkers identified here could also be developed to stratify cancer risk in humans that might be addressed by dietary alterations.

## Supplementary Information

Below is the link to the electronic supplementary material.

Supplementary file1 (PDF 679 KB)

## References

[CR1] Abo-Hashema KA, Cake MH, Power GW, Clarke D (1999). Evidence for triacylglycerol synthesis in the lumen of microsomes via a lipolysis-esterification pathway involving carnitine acyltransferases. Journal of Biological Chemistry.

[CR2] Alves da Silva AV, de Castro Oliveira SB, Di Rienzi SC, Brown-Steinke K, Dehan LM, Rood JK, Carreira VS, Le H, Maier EA, Betz KJ, Aihara E, Ley RE, Preidis GA, Shen L, Moore SR (2019). Murine methyl donor deficiency impairs early growth in association with dysmorphic small intestinal crypts and reduced gut microbial community diversity. Current Developments in Nutrition.

[CR3] Bao XR, Ong SE, Goldberger O, Peng J, Sharma R, Thompson DA, Vafai SB, Cox AG, Marutani E, Ichinose F, Goessling W, Regev A, Carr SA, Clish CB, Mootha VK (2016). Mitochondrial dysfunction remodels one-carbon metabolism in human cells. Elife.

[CR4] Bernstein C, Holubec H, Bhattacharyya AK, Nguyen H, Payne CM, Zaitlin B, Bernstein H (2011). Carcinogenicity of deoxycholate, a secondary bile acid. Archives of Toxicology.

[CR5] Bistulfi G, Vandette E, Matsui S, Smiraglia DJ (2010). Mild folate deficiency induces genetic and epigenetic instability and phenotype changes in prostate cancer cells. BMC Biology.

[CR6] Calviello G, Di Nicuolo F, Gragnoli S, Piccioni E, Serini S, Maggiano N, Tringali G, Navarra P, Ranelletti FO, Palozza P (2004). n-3 PUFAs reduce VEGF expression in human colon cancer cells modulating the COX-2/PGE2 induced ERK-1 and -2 and HIF-1alpha induction pathway. Carcinogenesis.

[CR7] Centers for Disease, C, & Prevention. (2004). Spina bifida and anencephaly before and after folic acid mandate--United States, 1995–1996 and 1999–2000. *Morbidity and Mortality Weekly Report, 53*(17), 362–365. https://www.ncbi.nlm.nih.gov/pubmed/15129193 15129193

[CR8] Chae YK, Yun JH (2007). Folic acid and prevention of colorectal adenomas. JAMA.

[CR9] Chong J, Wishart DS, Xia J (2019). Using MetaboAnalyst 4.0 for comprehensive and integrative metabolomics data analysis. Current Protocols in Bioinformatics.

[CR10] Cole BF, Baron JA, Sandler RS, Haile RW, Ahnen DJ, Bresalier RS, McKeown-Eyssen G, Summers RW, Rothstein RI, Burke CA, Snover DC, Church TR, Allen JI, Robertson DJ, Beck GJ, Bond JH, Byers T, Mandel JS, Mott LA, Pearson LH, Barry EL, Rees JR, Marcon N, Saibil F, Ueland PM, Greenberg ER, Polyp Prevention Study, G (2007). Folic acid for the prevention of colorectal adenomas: a randomized clinical trial. JAMA.

[CR11] Connors J, Dawe N, Van Limbergen J (2018). The role of succinate in the regulation of intestinal inflammation. Nutrients.

[CR12] Dalla Pozza E, Dando I, Pacchiana R, Liboi E, Scupoli MT, Donadelli M, Palmieri M (2020). Regulation of succinate dehydrogenase and role of succinate in cancer. Seminars in Cell & Developmental Biology.

[CR13] Dehaven CD, Evans AM, Dai H, Lawton KA (2010). Organization of GC/MS and LC/MS metabolomics data into chemical libraries. Journal of Cheminformatics.

[CR14] Evans AM, DeHaven CD, Barrett T, Mitchell M, Milgram E (2009). Integrated, nontargeted ultrahigh performance liquid chromatography/electrospray ionization tandem mass spectrometry platform for the identification and relative quantification of the small-molecule complement of biological systems. Analytical Chemistry.

[CR15] Fearon ER (2011). Molecular genetics of colorectal cancer. Annual Review of Pathology: Mechanisms of Disease.

[CR16] Fernandez ML, Vega-Lopez S (2005). Efficacy and safety of sitosterol in the management of blood cholesterol levels. Cardiovascular Drug Reviews.

[CR17] Friedman, J., Hastie, T., & Tibshirani, R. (2010). Regularization paths for generalized linear models via coordinate descent. *Journal of Statistical Software, 33*(1), 1–22. https://www.ncbi.nlm.nih.gov/pubmed/20808728 PMC2929880 20808728

[CR18] Giardina C, Nakanishi M, Khan A, Kuratnik A, Xu W, Brenner B, Rosenberg DW (2015). Regulation of VDR Expression in Apc-mutant mice, human colon cancers and adenomas. Cancer Prevention Research (Phila).

[CR19] Guo L, Milburn MV, Ryals JA, Lonergan SC, Mitchell MW, Wulff JE, Alexander DC, Evans AM, Bridgewater B, Miller L, Gonzalez-Garay ML, Caskey CT (2015). Plasma metabolomic profiles enhance precision medicine for volunteers of normal health. Proceedings of the National Academy of Sciences of the United States of America.

[CR20] Gylling B, Van Guelpen B, Schneede J, Hultdin J, Ueland PM, Hallmans G, Johansson I, Palmqvist R (2014). Low folate levels are associated with reduced risk of colorectal cancer in a population with low folate status. Cancer Epidemiology, Biomarkers & Prevention.

[CR21] Han J, Jackson D, Holm J, Turner K, Ashcraft P, Wang X, Cook B, Arning E, Genta RM, Venuprasad K, Souza RF, Sweetman L, Theiss AL (2018). Elevated d-2-hydroxyglutarate during colitis drives progression to colorectal cancer. Proceedings of the National Academy of Sciences of the United States of America.

[CR22] Hanley MP, Aladelokun O, Kadaveru K, Rosenberg DW (2020). Methyl donor deficiency blocks colorectal cancer development by affecting key metabolic pathways. Cancer Prevention Research (Phila).

[CR23] Hanley MP, Kadaveru K, Perret C, Giardina C, Rosenberg DW (2016). Dietary methyl donor depletion suppresses intestinal adenoma development. Cancer Prevention Research (Phila).

[CR24] Hanley MP, Rosenberg DW (2015). One-carbon metabolism and colorectal cancer: potential mechanisms of chemoprevention. Current Pharmacology Reports.

[CR25] Ikeda I, Sugano M (1983). Some aspects of mechanism of inhibition of cholesterol absorption by beta-sitosterol. Biochimica et Biophysica Acta.

[CR26] Jauhiainen A, Madhu B, Narita M, Narita M, Griffiths J, Tavare S (2014). Normalization of metabolomics data with applications to correlation maps. Bioinformatics.

[CR27] Kadaveru K, Protiva P, Greenspan EJ, Kim YI, Rosenberg DW (2012). Dietary methyl donor depletion protects against intestinal tumorigenesis in Apc(Min/+) mice. Cancer Prevention Research (Phila).

[CR28] Kim YI (2004). Folate, colorectal carcinogenesis, and DNA methylation: Lessons from animal studies. Environmental and Molecular Mutagenesis.

[CR29] Kim YI (2006). Folate: A magic bullet or a double edged sword for colorectal cancer prevention?. Gut.

[CR30] Kirpich A, Ainsworth EA, Wedow JM, Newman JRB, Michailidis G, McIntyre LM (2018). Variable selection in omics data: A practical evaluation of small sample sizes. PLoS One.

[CR31] Lawrance AK, Deng L, Rozen R (2009). Methylenetetrahydrofolate reductase deficiency and low dietary folate reduce tumorigenesis in Apc min/+ mice. Gut.

[CR32] Leichter J, Ottem A, Hahn P (1987). Does carnitine have a role in fat absorption?. Life Sci.

[CR33] Liesenfeld DB, Botma A, Habermann N, Toth R, Weigel C, Popanda O, Klika KD, Potter JD, Lampe JW, Ulrich CM (2016). Aspirin reduces plasma concentrations of the oncometabolite 2-hydroxyglutarate: results of a randomized, double-blind, crossover trial. Cancer Epidemiology, Biomarkers & Prevention.

[CR34] Mahmoud AM, Ali MM (2019). Methyl donor micronutrients that modify DNA methylation and cancer outcome. Nutrients.

[CR35] Miyo M, Konno M, Colvin H, Nishida N, Koseki J, Kawamoto K, Tsunekuni K, Nishimura J, Hata T, Takemasa I, Mizushima T, Doki Y, Mori M, Ishii H (2017). The importance of mitochondrial folate enzymes in human colorectal cancer. Oncology Reports.

[CR36] Moser, A. R., Luongo, C., Gould, K. A., McNeley, M. K., Shoemaker, A. R., & Dove, W. F. (1995). ApcMin: a mouse model for intestinal and mammary tumorigenesis. *European Joural of Cancer, 31A*(7–8), 1061–1064. https://www.ncbi.nlm.nih.gov/pubmed/7576992 10.1016/0959-8049(95)00181-h 7576992

[CR37] Noguchi K, Konno M, Koseki J, Nishida N, Kawamoto K, Yamada D, Asaoka T, Noda T, Wada H, Gotoh K, Sakai D, Kudo T, Satoh T, Eguchi H, Doki Y, Mori M, Ishii H (2018). The mitochondrial one-carbon metabolic pathway is associated with patient survival in pancreatic cancer. Oncology Letters.

[CR38] Rocchiccioli F, Aubourg P, Bougneres PF (1986). Medium- and long-chain dicarboxylic aciduria in patients with Zellweger syndrome and neonatal adrenoleukodystrophy. Pediatric Research.

[CR39] Ryan BM, Weir DG (2001). Relevance of folate metabolism in the pathogenesis of colorectal cancer. Journal of Laboratory and Clinical Medicine.

[CR40] Scheppach W (1994). Effects of short chain fatty acids on gut morphology and function. Gut.

[CR41] Song, J., Medline, A., Mason, J. B., Gallinger, S., & Kim, Y. I. (2000). Effects of dietary folate on intestinal tumorigenesis in the apcMin mouse. *Cancer Research, 60*(19), 5434–5440. https://www.ncbi.nlm.nih.gov/pubmed/11034085 11034085

[CR42] Suzuki H, Yamada J, Watanabe T, Suga T (1989). Compartmentation of dicarboxylic acid beta-oxidation in rat liver: Importance of peroxisomes in the metabolism of dicarboxylic acids. Biochimica et Biophysica Acta.

[CR43] van den Berg RA, Hoefsloot HC, Westerhuis JA, Smilde AK, van der Werf MJ (2006). Centering, scaling, and transformations: improving the biological information content of metabolomics data. BMC Genomics.

[CR44] Van Guelpen B, Hultdin J, Johansson I, Hallmans G, Stenling R, Riboli E, Winkvist A, Palmqvist R (2006). Low folate levels may protect against colorectal cancer. Gut.

[CR45] Wang D, Dubois RN (2006). Prostaglandins and cancer. Gut.

[CR46] Washington L, Cook GA, Mansbach CM (2003). Inhibition of carnitine palmitoyltransferase in the rat small intestine reduces export of triacylglycerol into the lymph. Journal of Lipid Research.

[CR47] Zhao, T., Mu, X., You, Q. (2017). Succinate: An initiator in tumorigenesis and progression. *Oncotarget, 8*(32), 53819–53828. 10.18632/oncotarget.17734 10.18632/oncotarget.17734 PMC5581152 28881853

[CR48] Zou, H., & Hastie, T. (2005). Regularization and variable selection via the elastic net. *Journal of the royal statistical society: series B (statistical methodology, 67.2* 301–320. https://web.stanford.edu/~hastie/Papers/B67.2%20(2005)%20301-320%20Zou%20&%20Hastie.pdf

