# Supplementary material for: Fatty acid metabolism and colon cancer protection by dietary methyl donor restriction
Source: Metabolomics. 2021 Sep 3;17(9):80. doi: 10.1007/s11306-021-01831-1 (PMC8416812; doi:10.1007/s11306-021-01831-1)
Supplement: Supplementary file 1 — Supplementary file1 (PDF 679 KB) [file 11306_2021_1831_MOESM1_ESM.pdf]

Fatty acid metabolism and colon cancer protection by dietary methyl donor restriction

Oladimeji Aladelokun[1]*, Matthew Hanley[1], Jinjian Mu[2], John C. Giardina[3], Daniel W. Rosenberg[1], Charles Giardina[4]

[1]Center for Molecular Oncology, University of Connecticut Health Center, Farmington, Connecticut.

[2]Statistical Consulting Services, University of Connecticut, Storrs, Connecticut.

[3]Center for Health Decision Science, Harvard T.H. Chan School of Public Health, Massachusetts.

[4]Department of Molecular and Cellular Biology, University of Connecticut, Storrs, Connecticut

Running Title: Modulation of lipid metabolism by dietary methyl donors and CRC development

***Corresponding Author:** Oladimeji Aladelokun, The University of Connecticut School of Medicine 263 Farmington Ave., Farmington, CT 06030-3101. Email: oladimeji.aladelokun@uconn.edu

Supplementary Figure 1: MDD alters levels of metabolites involved in one carbon metabolism.

MDD diet caused a significant reduction in the level of: (A) dimethylglycine (-6.0, $p < 0.0001$), and (B) cystine (-4.0, $p < 0.0001$). All groups marked with “a” have a statistically significant difference compared to groups marked with “b”.

Supplementary Table 1: A list of 33 metabolites identified as significant predictors of tumor count using elastic net analysis.

Supplementary Figure 2: Impact of MDD on COX2/mPGES-1 expression in colon cancer.

(a) Representative image from immunohistochemistry (IHC) for mPGES-1 reveals that MDD causes a reduced expression of mPGES-1. *Note the absence of mPGES-1 positive cells within the MDD colon section.* (b) Similarly, mRNA levels of *mPGES-1* was downregulated in normal mucosa (-2.6-fold) and colon tumors (-2.1-fold) under conditions of methyl donor restriction. Elevated

mPGES-1 in the MDS control mice suggests an accelerated production of prostaglandins, known to be pro-tumorigenic. (c) Downregulation of *COX2* mRNA level in the normal mucosa (-1.5-fold) and colon tumors (-5.0-fold) is noticeable after MDD, implicating the arachidonic acid-metabolizing enzyme as a mediator in colon tumorigenesis, a pathway potentially inhibited by MDD. These figures were generated from a conditional Apc mouse (*Cdx2-Cre*^{ERT2}*;Apcfl/fl)* on the MDD and MDS diets. Mice were treated with three consecutive daily doses of tamoxifen (100 mg/kg). Three weeks after tamoxifen treatment, mice were euthanized and colon tissue was prepared for IHC or RNA analysis. IHC was performed using mPGES1 antibody (Cayman,Catalog #160140). RNA was quantified by Qubit and used to synthesize cDNA (iTaq kit) and analyzed by real-time PCR using SYBR green (BioRad). Primers were designed using Primer3 software. Statistical significance was assessed by one-way analysis of variance (ANOVA). All groups marked with “a” have a statistically significant difference compared to groups marked with “b” or “c” or “d”. Similarly, all groups marked with “b” have a significantly significant difference compared to groups marked with “a”, “c” or “d”.

Supplementary Figure 3: Box plots of metabolites identified as significant predictors of tumor count using the elastic net analysis. These metabolites failed normality tests, so comparisons were done using nonparametric MANOVA test. Overall metabolites levels differed significantly between diet groups (p = 0.01). Bonferroni correction to adjust for multiple comparisons (6 hypotheses were tested with α = 0.05).

Supplementary Figure 4: Box plots of metabolites identified as significant predictors of tumor count using elastic net analysis.

Supplemental Figure 1

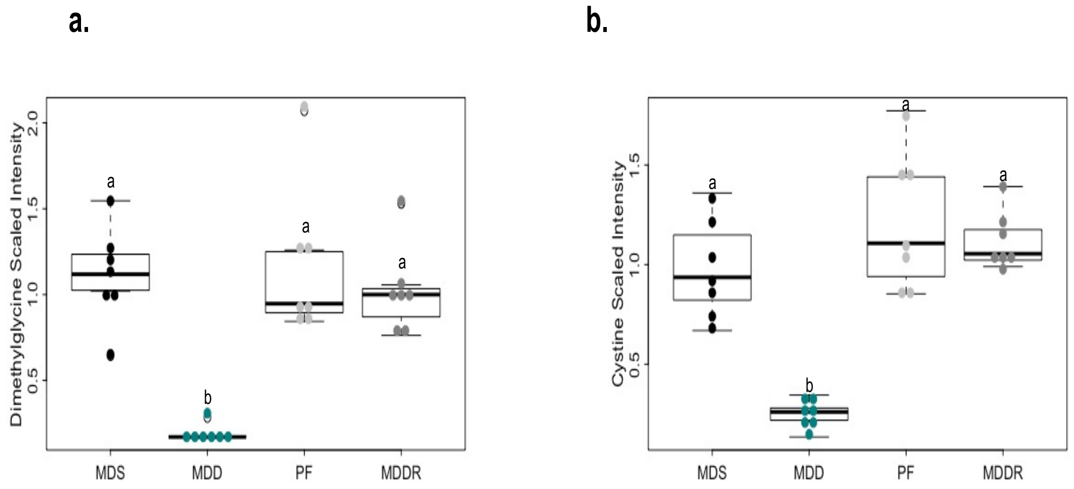

Supplementary Table 1: Metabolites identified as significant predictors of tumor count using elastic net.

Metabolites	Coefficient	MSI level*
Azelate	0.0878	1
7 α-hydroxycholesterol	0.0865	1
Pyrophosphate	0.0447	1
Vitamin B12	0.0324	1
Taurine	0.0268	1
5-Hydroxyindoleacetate	0.0250	1
1-Oleoylglycerophosphoethanolamine	0.0227	1
Arachidonate	0.022	1

Metabolites	Coefficient	MSI level*
4-Hydroxyphenylacetate	0.0168	1
12-HETE	0.0074	1
Sucrose	0.0052	1
Guanosine	0.0028	1
7 β-hydroxycholesterol	0.0024	1
3-Sulfooxyphenylpropanoic Acid	0.0017	1
Mannitol	-0.0010	1
Vitamin C	-0.0030	1
2-monolinolein	-0.0033	1
Valerylcarnitine	-0.0036	1
Citrate	-0.0045	1
Cholestanol	-0.0052	1
Indolelactate	-0.0053	1
Xylitol	-0.0066	1
Valerylglycine	-0.009	1
Urate	-0.0153	1
2-Hydroxybutyrate	-0.0168	1
Isobutyrylcarnitine	-0.0203	1

**Metabolites**	**Coefficient**	**MSI level***
Beta_hydroxypyruvate	-0.0212	1
Pro_hydroxy_pro	-0.0244	1
3 ureidopropionate	-0.0323	1
Creatinine	-0.0364	1
Methylbutyrylcarnitine	-0.0390	1
β-sitosterol	-0.0864	1
Succinate	-0.0959	1

*Metabolomics standards initiative (MSI) level is based on MSI guidelines for naming non-novel metabolites (Sumner et al., 2007). Selected metabolites in this dataset are classified as “Tier 1”.

Supplemental Figure 2

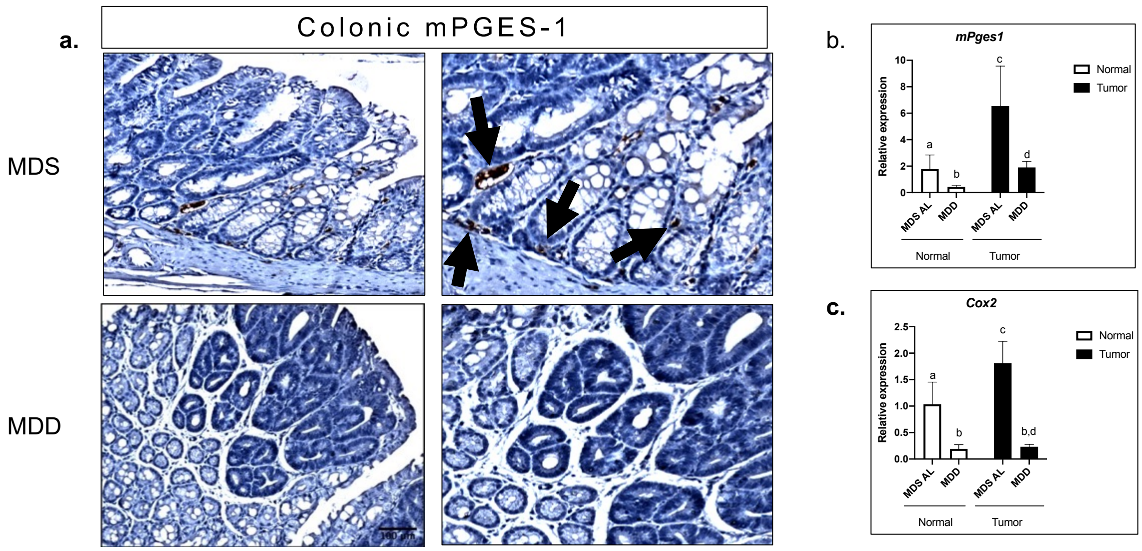

Supplemental Figure 3

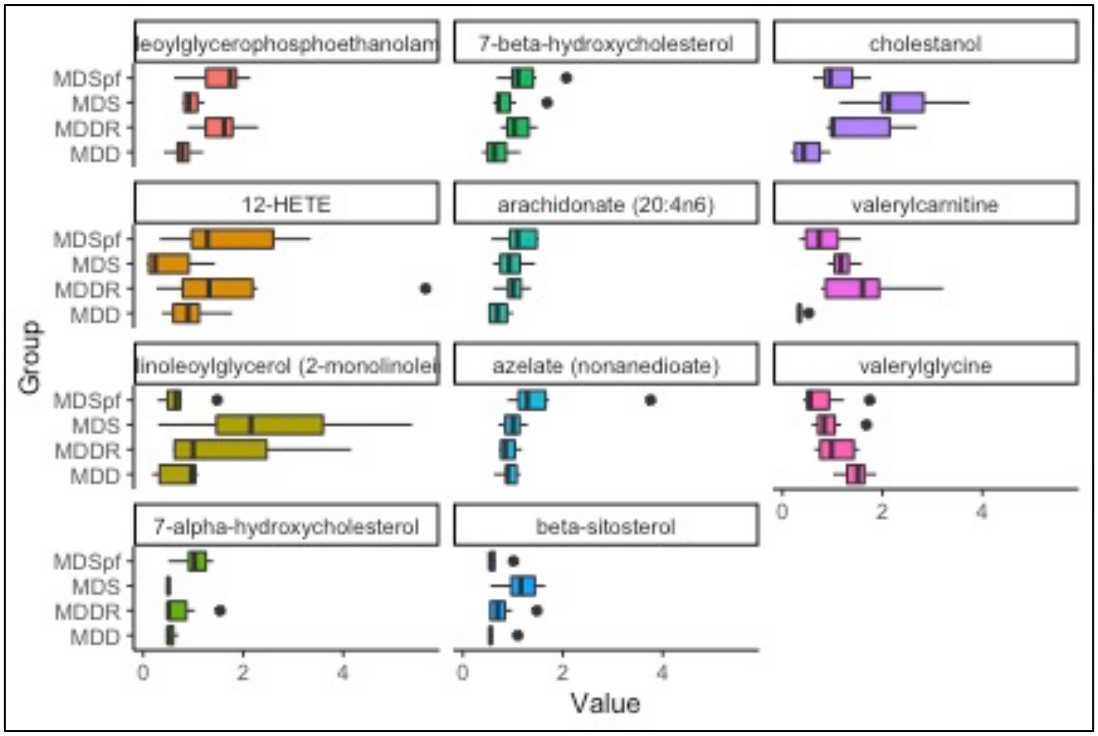

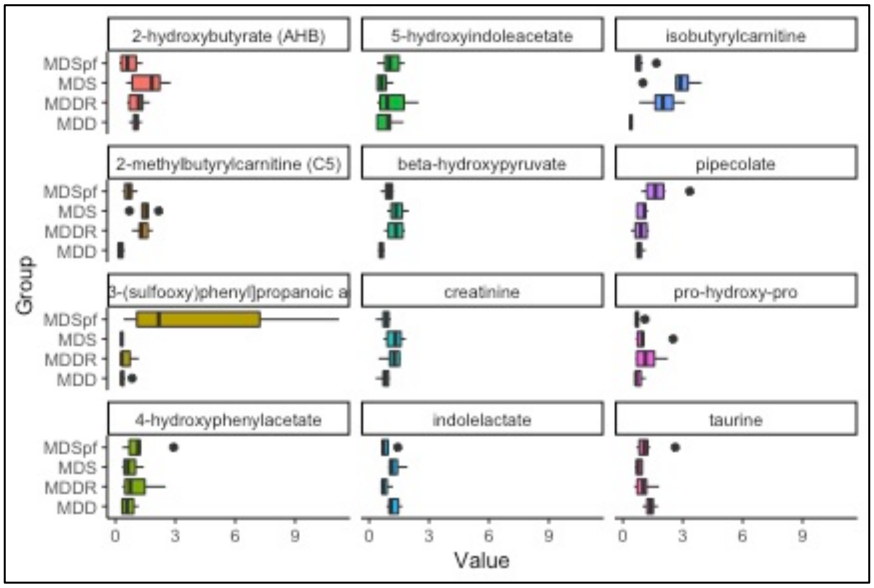
2-hydroxybutyrate (AHB)
5-hydroxyindoleacetate
isobutyrylcarnitine
2-methylbutyrylcarnitine (C5)
beta-hydroxypyruvate
pipecolate
3-(sulfooxy)phenyl]propanoic a
creatinine
pro-hydroxy-pro
4-hydroxyphenylacetate
indolelactate
taurine
MDSpf
MDS
MDDR
MDD
Group
Value

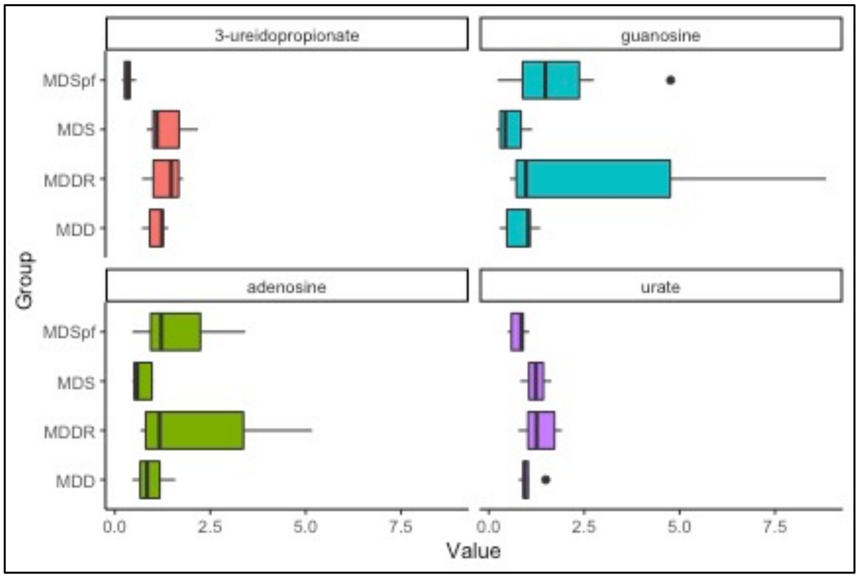
3-ureidopropionate
guanosine
adenosine
urate
MDSpf
MDS
MDDR
MDD
Group
Value

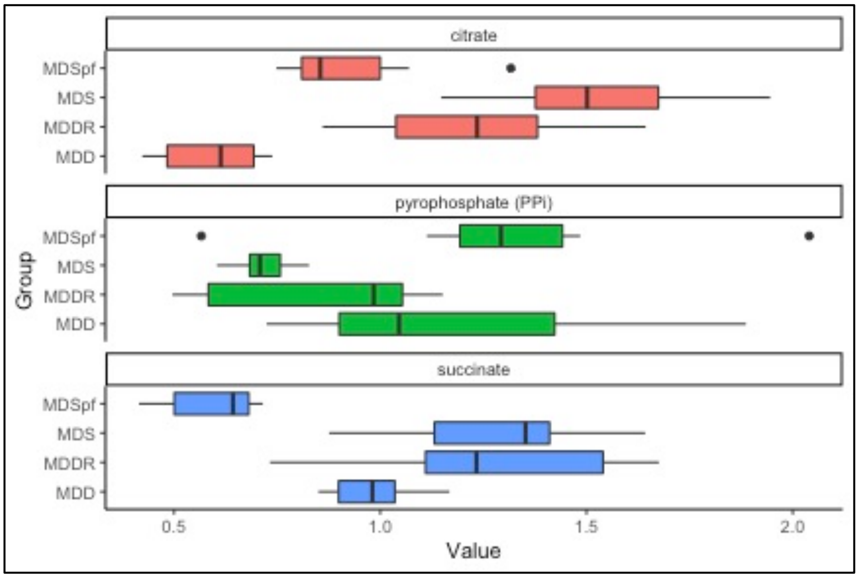
citrate
pyrophosphate (PPi)
succinate
MDSpf
MDS
MDDR
MDD
Group
Value

Supplementary Figure 4

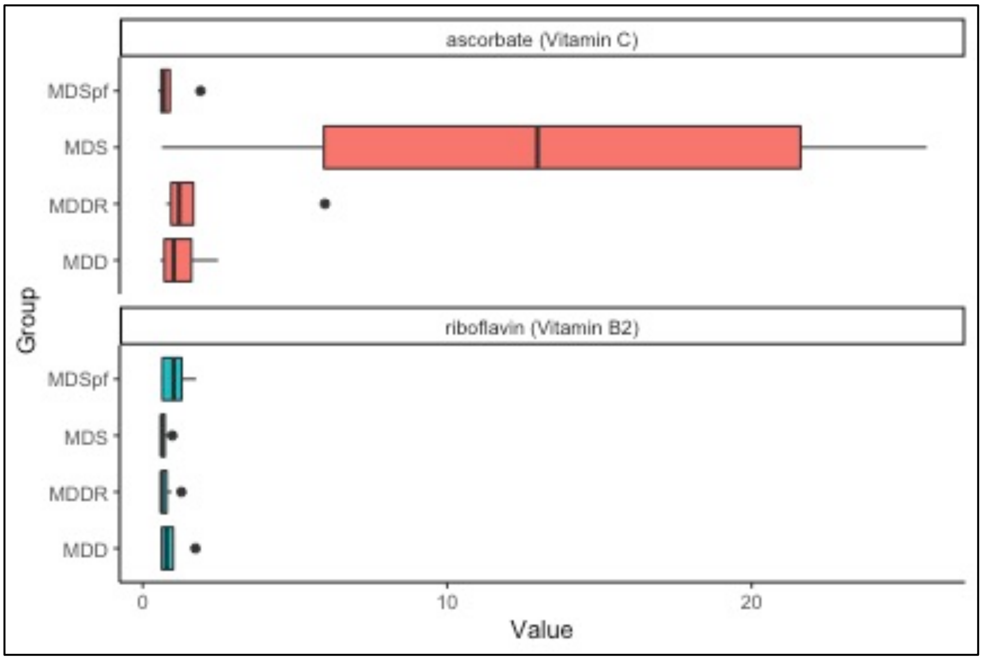

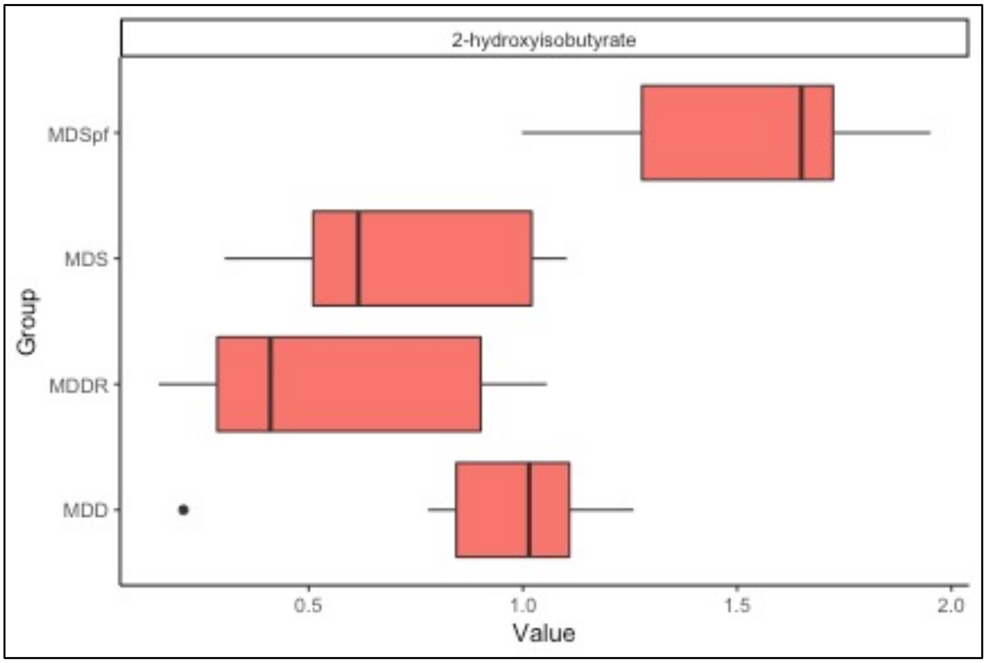

Supplementary Table 2: Composition of MDS and MDD diets

Component	MDS diet (g/kg)	MDD diet (g/kg)
L-Alanine	3.5	3.5
L- Arginine HCl	12.1	12.1
L-Asparagine	6	6
L-Aspartic acid	3.5	3.5
L-Cystine	3.5	3.5
L-Glutamic Acid	40.0	40.0
Glycine	23.3	23.3
L-Histidine HCl, monohydrate	4.5	4.5
L-Isoleucine	8.2	8.2
L-Leucine	11.1	11.1
L-Lysine HCl	18.0	18.0
**L-Methionine**	**8.2**	**0**
L-Phenylalanine	7.5	7.5
L-Proline	3.5	3.5
L-Serine	3.5	3.5
L-Threonine	8.2	8.2
L-Tryptophan	1.8	1.8
L-Tyrosine	5.0	5.0
L-Valine	8.2	8.2
Sucrose	351.68	351.68
Corn Starch	150.0	150.0
Maltodextrin	150.0	150.0
Soybean Oil	80	80
Cellulose	30.0	30.0
Mineral Mix, AIN-93M-MX	35.0	35.0
Calcium Phosphate, monobasic, monohydrate	8.2	8.2
Vitamin Mix, AIN-93-VX	13.0	13.0
**Choline Bitartrate**	**2.5**	**0**
Niacin	0.039	0.039
Calcium Pantothenate	0.0208	0.0208
Pyridoxine HCl	0.0091	0.0091
Thiamin HCl	0.0078	0.0078
Riboflavin	0.0078	0.0078
**Folic Acid**	**0.0026**	**0**
TBHQ, antioxidant	0.02	0.02
Biotin	0.0003	0.0003
**Vitamin B12 (0.1% in mannitol)**	**0.0325**	**0**
Vitamin E DL-alpha tocopheryl acetate	0.195	0.195
Vitamin A Palmitate	0.0104	0.0104
Vitamin D3 (cholecalciferol)	0.0026	0.0026
Vitamin K12 (phylloquinone)	0.001	0.001
DL-Homocystine	0	9

Reference

Sumner, L. W., Amberg, A., Barrett, D., Beale, M. H., Beger, R., Daykin, C. A., Fan, T. W., Fiehn, O., Goodacre, R., Griffin, J. L., Hankemeier, T., Hardy, N., Harnly, J., Higashi, R., Kopka, J., Lane, A. N., Lindon, J. C., Marriott, P., Nicholls, A. W., Reily, M. D., Thaden, J. J., & Viant, M. R. (2007, Sep). Proposed minimum reporting standards for chemical analysis Chemical Analysis Working Group (CAWG) Metabolomics Standards Initiative (MSI). *Metabolomics, 3*(3), 211-221. https://doi.org/10.1007/s11306-007-0082-2
